# Supplementary material for: Isobutanol production freed from biological limits using synthetic biochemistry
Source: Nat Commun. 2020 Aug 27;11:4292. doi: 10.1038/s41467-020-18124-1 (PMC7453195; doi:10.1038/s41467-020-18124-1)
Supplement: Supplementary file 1 — Supplementary Information [file 41467_2020_18124_MOESM1_ESM.pdf]

Isobutanol production freed from biological limits using synthetic biochemistry

Sherkhanov *et al.*

Supplementary Table 1. Summary of production parameters obtained using cell-based methods.

Organism	Substrate	Titer (g/L)	Yield (%)	Productivity (g/L/h)	Comments	Ref.
Magnusiomyces magnusii	Glucose and 2-oxoisovalerate	0.76	nd	0.01*		1
Saccharomyces cerevisiae	Glucose	0.26	nd	0.003		2
Saccharomyces cerevisiae	Xylose	3.1	9.4	0.04	fed-batch fermentation	3
Saccharomyces cerevisiae	Glucose	8.4	13	0.04*	Fed-batch fermentation	4
Saccharomyces cerevisiae	Glucose	0.15	nd	nd		5
Saccharomyces cerevisiae	Glucose	0.64	1.6*	nd		6
Saccharomyces cerevisiae	Glucose	1.62	3.9*	0.25*		7
Saccharomyces cerevisiae	Glucose	0.68	8.3*	nd		8
Saccharomyces cerevisiae	xylose	0.09	nd	0.002*		9
Clostridium thermocellum	cellulose	5.1	nd	nd		10
Clostridium thermocellum	cellulose	5.4	41	0.07*		11
Clostridium cellulolyticum	cellulose	0.66	nd	nd		12
Geobacillus thermoglucosidasius	cellobiose	0.6	8.3	0.01*		13
Bacillus subtilis	Glucose	6.1	63	0.17*	fed-batch fermentation	14
Bacillus subtilis	Aminoacids	0.09	47	nd		15
Bacillus megaterium	2-ketoisovalerate	0.3	80	0.01*	cultures grown and product extracted under supercritical carbon dioxide	16
Synechococcus elongatus	carbon dioxide	0.45	nd	0.003*		17
Ralstonia eutropha	carbon dioxide	0.85	nd	nd	bioreactor	18
Escherichia coli	Glucose	50	68	0.7	bioreactor with *in situ* product removal (gas stripping)	19
Escherichia coli	Glucose	22	86	0.31*		20
Escherichia coli	Aminoacids	2	56	0.02*		21

nd – not determined
* - the values were not reported but were estimated from graphs in the publication if available.

Supplementary Table 2. Summary of genome mining screen.

	Enzyme	Organism	Accession number	Codon optimized?	Expression level	Activity at 25 °C	Isobutanol stability
Hex	Hexokinase	*T. maritima*	AAD36537	No	+++	+++	+++
Glk	Glucokinase	*G. stearothermophilus*	KOR95707	No	+++	++++	
Pgi	Glucose-6-phosphate isomerase	*T. maritima*	*AAD36455*	No	+++	+++	+++
	Glucose-6-phosphate isomerase	*P. aerophilum*	*HII46470*	No	+	+	
Pfk	Phosphofructokinase B	*E. coli*	*ACT43547*	No	+++	++++	++
	Phosphofructokinase A	*G. stearothermophilus*	*KOR92562*	No	+++	++++	-
	Phosphofructokinase	*P. aerophilum*	*HII48166*	No	+	+	-
	Phosphofructokinase	*T. thermophilus*	*BAD71785*	No	++	++	
Fba	Fructose-1,6-bisphosphate aldolase	*T. maritima*	*AAD35362*	No	+	+	+++
	Fructose-1,6-bisphosphate aldolase	*G. stearothermophilus*	*KOR93738*	No	+++	++	
	Fructose-1,6-bisphosphate aldolase	*T. thermophilus*	*BAD71596*	No	++	++	
Tpi-Pgk	Triose Phosphate Isomerase - Phosphoglycerate Kinase Fusion	*T. maritima*	*AAD35771*	No	+++	+++	+++
GapN	Glyceraldehyde-3-phosphate Dehydrogenasnon-phosphorylating)	*T. kodakarensis*	*BAD84894*	No	++	++	+++
	Glyceraldehyde-3-phosphate Dehydrogenasnon-phosphorylating)	*T. tenax*	*CCC81810*	No	++	+	
	Glyceraldehyde-3-phosphate Dehydrogenasnon-phosphorylating)	*S. solfataricus*	*AAK43292*	No	++	+	
	Glyceraldehyde-3-phosphate Dehydrogenasnon-phosphorylating)	*A. pernix*	*BAA80789*	No	-	-	
	Glyceraldehyde-3-phosphate Dehydrogenasnon-phosphorylating)	*M. jannaschii*	*AAB99418*	No	++	-	
	Glyceraldehyde-3-phosphate Dehydrogenasnon-phosphorylating)	*T. takaii*	*BAT70977*	No	++	-	
	Glyceraldehyde-3-phosphate Dehydrogenasnon-phosphorylating)	*M. sedula*	*ABP95456*	No	++	-	
	Glyceraldehyde-3-phosphate Dehydrogenasnon-phosphorylating)	*P. furiosus*	*AAL80879*	No	-	-	
	Glyceraldehyde-3-phosphate Dehydrogenasnon-phosphorylating)	*T. neapolitana*	*ACM23724*	No	-	-	
GapDH	Glyceraldehyde-3-phosphate Dehydrogenasphosphorylating)	*A. fulgidus*	*AAB89515*	No	++	++	+++
	Glyceraldehyde-3-phosphate Dehydrogenasphosphorylating)	*G. stearothermophilus*	*KOR95271*	No	+++	+++	
Pgk	Phosphoglycerate Kinase	*T. maritima*	*AAD35771*	No	+++	+++	+++
	Phosphoglycerate Kinase	*G. stearothermophilus*	*KOR95272*	No	+++	+++	
GpmA	Phosphoglycerate Mutase (2,3-bisphosphoglycerate dependent)	*E. coli*	*ACT42583*	No	+++	+++	-
GpmI	Phosphoglycerate Mutase (2,3-bisphosphoglycerate independent)	*G. stearothermophilus*	*KOR95229*	No	+++	+++	+++
Eno	Phosphoenolpyruvate hydratase	*A. fulgidus*	*AIG98006*	No	++	++	
	Phosphoenolpyruvate hydratase	*T.thermophilus*	*BAD69825*	No	++	++	+++
	Phosphoenolpyruvate hydratase	*G. stearothermophilus*	*KOR95275*	No	+++	+++	

Pyk	Pyruvate Kinase	*T. maritima*	*AAD35300*	No	++	++	
	Pyruvate Kinase	*T.thermophilus*	*BAD69826*	No	++	++	+++
	Pyruvate Kinase A	*G. stearothermophilus*	*KOR92563*	No	+++	+++	
IlvC	Ketol-acid Reductoisomerase	*T. thermophilus*	*BAD71034*	Yes	++	+	+++
	Ketol-acid Reductoisomerase	*G. stearothermophilus*	*KOR94737*	Yes	+++	++	+++
	Ketol-acid Reductoisomerase	*Thermococcus chitonophagus*	*ASJ16613*	Yes	+++	++	++
	Ketol-acid Reductoisomerase	*Caldicellulosiruptor bescii* DSM 6725	*ACM59630*	Yes	+	+	+++
IlvD	Dihydroxyacid Dehydratase	*S. mutans*	*AJD56310*	No	+++	++	++
	Dihydroxyacid Dehydratase	*G. stearothermophilus*	*KOR95295*	No	+++	O_2 sensitive	
	Dihydroxyacid Dehydratase	*T. thermophilus*	*BAD71057*	No	++	O_2 sensitive	

Supplementary Table 3. Data collection and refinement statistics.

PDB identification number	6VGS
**Ligand bound**	Thiamine pyrophosphate (TTP) with Mg ion
**Data collection temperature (K)**	100
**Data Collection Statistics**	
**Resolution (Å, high limit)**	1.80
**Resolution (Å, low limit)**	100.00
**Space group**	$P2_12_12_1$
**Unit cell parameters**	
**a (Å)**	128.20
**b (Å)**	128.27
**c (Å)**	147.74
**α, β, γ (^{o})**	90.00, 90.00, 90.00
**Radiation source**	APS 24-ID-C
**Radiation wavelength (Å)**	0.97930
**Measured reflections**	1,972,360
**Unique reflections**	223,094
**Overall completeness (%)**	99.40
**Last shell completeness (%) (1.80-1.85 Å)**	97.20
**Overall redundancy**	8.8
**Last shell redundancy**	7.9
**Overall R_{merge}[a]**	0.151
**Last shell R_{merge} (1.80-1.85 Å)**	1.055
**Overall I/σ(I)**	12.3
**Last shell I/σ(I) (1.80-1.85 Å)**	2.1
**Refinement Statistics**	
**R_{work}[b]**	18.3
**R_{free}[c]**	21.0
**Number of residues (protein/water)**	2164/1181
**RMSD bond lengths (Å)**	0.008
**RMSD bond angles (^{o})**	1.423

[a] $R_{merge}(I) = \Sigma_{hkl} \Sigma_i |I_i(hkl) - <I(hkl)>| / \Sigma_{hkl} \Sigma_i I_i(hkl)$

[b] $R_{work} = \Sigma_{hkl} ||F_{obs}| - |F_{calc}|| / \Sigma_{hkl} |F_{obs}|$

[c] $R_{free} = \Sigma_{hkl} ||F_{obs}| - |F_{calc}|| / \Sigma_{hkl} |F_{obs}|$, where all reflections belong to a test set of 5% randomly selected data

Supplementary Table 4. Enzyme assay conditions.

	Coupling enzymes*	Cofactors and substrates
**TmHex**	PK/LDH	1 mM ATP, 2.5 mM PEP, 1 mM NADH, 3 mM Glucose
**TmPgi**	EcPfkB, PK/LDH	1 mM ATP, 2.5 mM PEP, 1 mM NADH, 3 mM Glucose-6-Phosphate
**EcPfkB**	PK/LDH	1 mM ATP, 2.5 mM PEP, 1 mM NADH, 3 mM Fructose-6-Phosphate
**TtFba**	TmTpi, TkGapN	1 mM $NADP^+$, 0.75 mM Glucose-1-Phosphate, 2.5 mM Fructose 1,6-bis-Phosphate
**TmTpi**	TkGapN	1 mM $NADP^+$, 0.75 mM Glucose-1-Phosphate, 2.5 mM Dihydroxyacetone Phosphate
**TkGapN**	TmTpi	1 mM $NADP^+$, 0.75 mM Glucose-1-Phosphate, 2.5 mM Dihydroxyacetone Phosphate
**AfGapDH**	TmTpi, TmPgk	1 mM $NADP^+$, 2.5 mM ADP, 4 mM PO_4, 2.5 mM Dihydroxyacetone Phosphate
**TmPgk**	PK/LDH	1 mM ATP, 1 mM NADH, 2.5 mM PEP, 2.5 mM Glycerate 3-Phosphate
**Gs iPgm**	TtEno, PK/LDH	2.5 mM ADP, 2 mM NADH, 4 mM PO_4, 0.5 mM $MnCl_2$, 2.5 mM Glycerate 3-Phosphate
**TtEno**	Gs iPgm, PK/LDH	2.5 mM ADP, 2 mM NADH, 4 mM PO_4, 0.5 mM $MnCl_2$, 2.5 mM Glycerate 3-Phosphate
**TtPyk**	LDH	1.5 mM ADP, 1 mM NADH, 3 mM PO_4, 1 mM Fructose 1,6-bis-Phosphate, 3 mM PEP
**AlsS**	GsIlvC	1 mM NADPH, 1 mM TPP, 5 mM Pyruvate
**GsIlvC**	AlsS	1 mM NADPH, 1 mM TPP, 5 mM Pyruvate
**SmIlvD**	KivD, EcYahK	1 mM NADPH, 1 mM TPP, 2.5 mM (R)-2,3-dihydroxy-isovalerate
**KivD**	EcYahK	1 mM NADPH, 1 mM TPP, 5 mM 2-ketoisovalerate
**EcYahK**		1 mM NADPH, 5 mM Isobutyraldehyde

*Enzyme concentrations given in Supplementary Table 5.

Supplementary Table 5. Enzyme amounts per assay.

Enzyme assayed	Amount added (µg)	Coupling enzymes amount added
**TmHex**	0.49	PK/LDH* (2 µL)
**TmPgi**	0.55	EcPfkB (76 µg)/PK/LDH (2 µL)
**EcPfkB**	1.52	PK/LDH (2 µL)
**TtFba**	10.47	TmTpi (43.15 µg)/TkGapN' (330 µg)
**TmTpi**	0.48	TkGapN' (114 µg)
**TkGapN'**	1.66	TmTpi (43.15 µg)
**AfGapDH**	122.2	TmTpi (43.15 µg)/TmPgk (390 µg)
**TmPgk**	1.56	PK/LDH (30 µL)
**Gs iPgm**	3.53	TtEno (216 µg)/PK/LDH (10 µL)
**TtEno**	1.08	Gs iPgm (348.25 µg)/PK/LDH (10 µL)
**TtPyk**	1.67	LDH** (2 µL)
**AlsS-P**	1.10	GsIlvC (183 µg)
**GsIlvC**	12.2	AlsS-P (169 µg)
**SmIlvD**	42.7	KivD-S (155 µg)/EcYahK (271 µg)
**KivD-S**	2.64	EcYahK (271 µg)
**EcYahK**	84	none

* PK/LDH solution was purchased from Sigma-Aldrich (catalog number: P0294)
** LDH was purchased from Millipore Sigma and resuspended in 50% glycerol solution containing 10 mM HEPES, pH 7.0, 100 mM KCl and 0.1 mM EDTA at a concentration of 0.2 units/µL (catalog number: 427217)

Supplementary Table 6. Enzyme specific activities and loadings for isobutanol production system.

Enzyme	Stock (mg/mL)	Activity (units/mg)	Loading concentration (mg/mL)	Units added (per mL)
TmHex	4.87	14.5±0.3	0.14	2.03
TmPgi	6.08	29.6±1.1	0.30	8.82
EcPfkB	13.47	8.3±0.1	0.22	1.82
TtFba	2.59	4.7±0.1	0.35	1.64
TmTpi	2.85	210±0.7	0.14	30.44
TkGapN'	9.73	7.6±0.8	0.46	3.45
AfGapDH	8.87	10.9±0.6	0.05	0.53
TmPgk	2.23	8.4±0.5	0.20	1.68
Gs iPgm	13.93	49.4±2.2	0.18	8.88
TtEno	6.75	117±5	0.66	77.18
TtPyk	4.82	23.8±0.8	0.20	4.76
BsAlsS-P	7.49	4.9±0.2	0.10	0.50
GsIlvC	9.86	0.9±0.1	1.13	1.01
SmIlvD	4.27	2.7±0.2	0.46	1.27
KivD-S	45.73	18.0±1.6	0.15	2.70
EcYahK	17.29	4.5±0.4	0.27	1.20

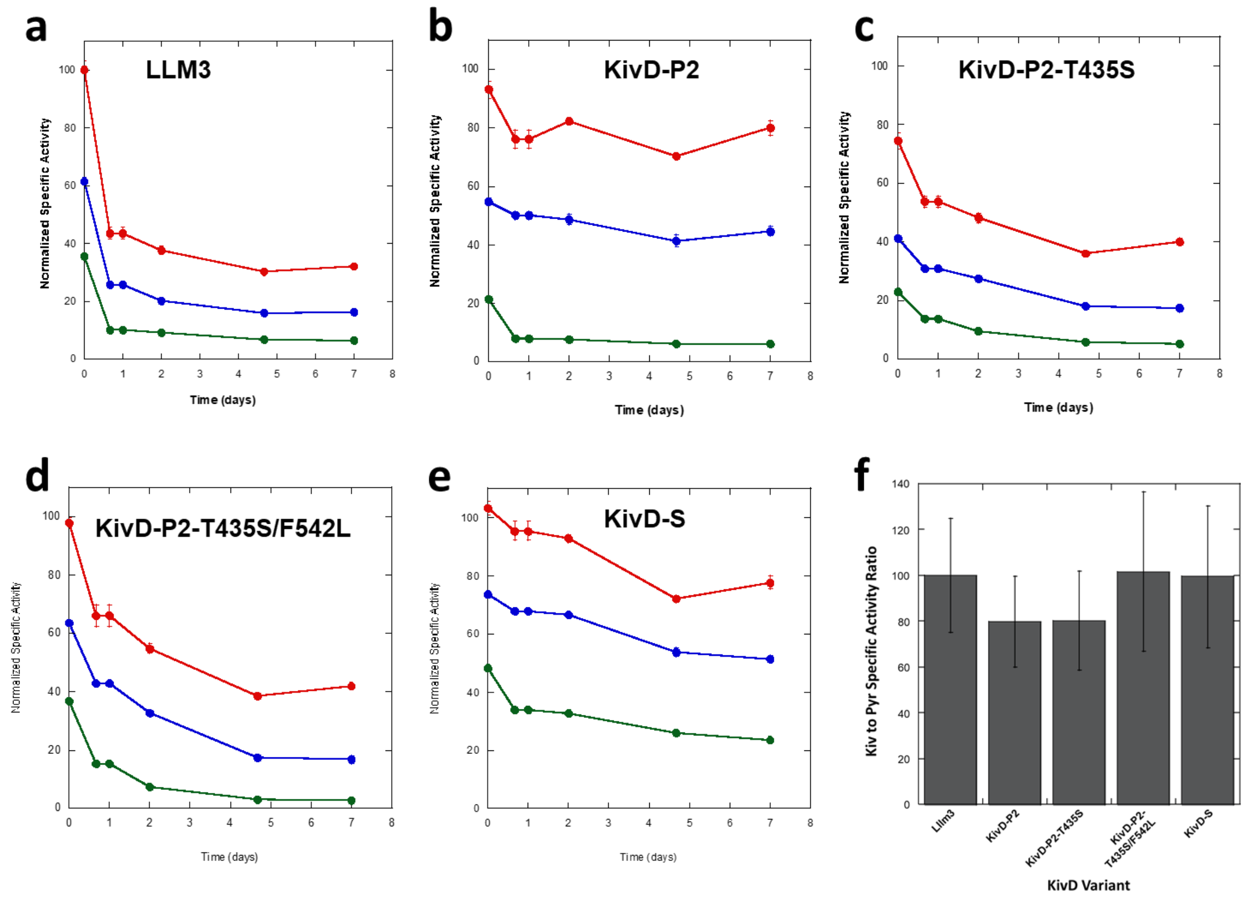

Supplementary Fig. 1. Stabilizing KivD. **a-e,** The activity of the indicated enzymes over time during incubation in 0% (red), 4% (blue) or 8% (green) isobutanol. In these screening assays, the enzyme solution was only diluted 2-fold in the final assay mix. Hence, the final assays contained 0%, 2% or 4% isobutanol, which accounts for the different initial activities. **f,** Ratio of specific activity for the substrates α-ketoisovalerate and pyruvate with the indicated enzymes. Errror bars represent standard deviations of biological triplicates. Source data are provided as a Source Data file.

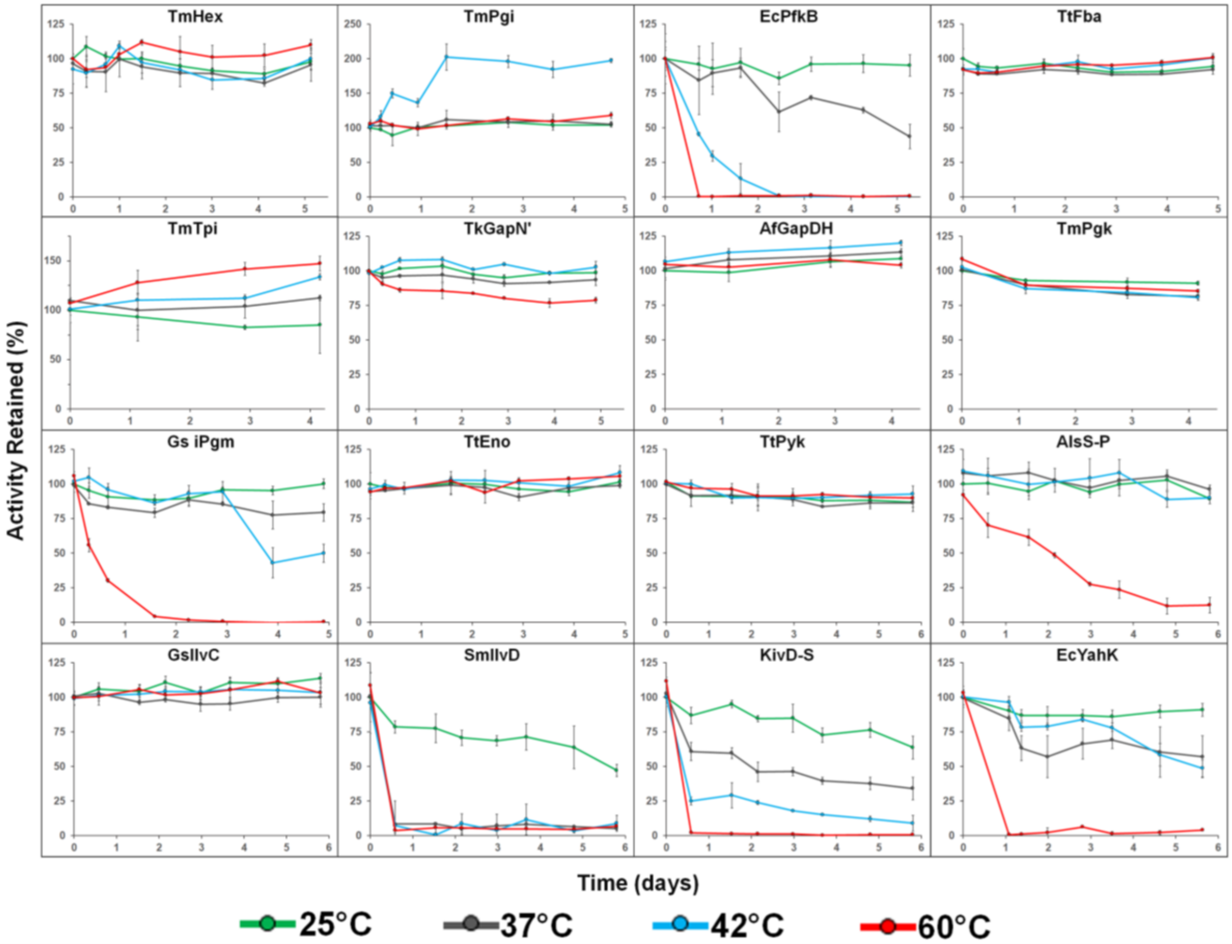

Supplementary Fig. 2. Thermal tolerance of enzymes used in this work. Enzymes were incubated at the indicated temperatures and aliquots assayed over time. Activity retained is the percentage of activity remaining compared to the activity at time zero and 25°C. Error bars represent the standard deviation of biological triplicates. Source data are provided as a Source Data file.

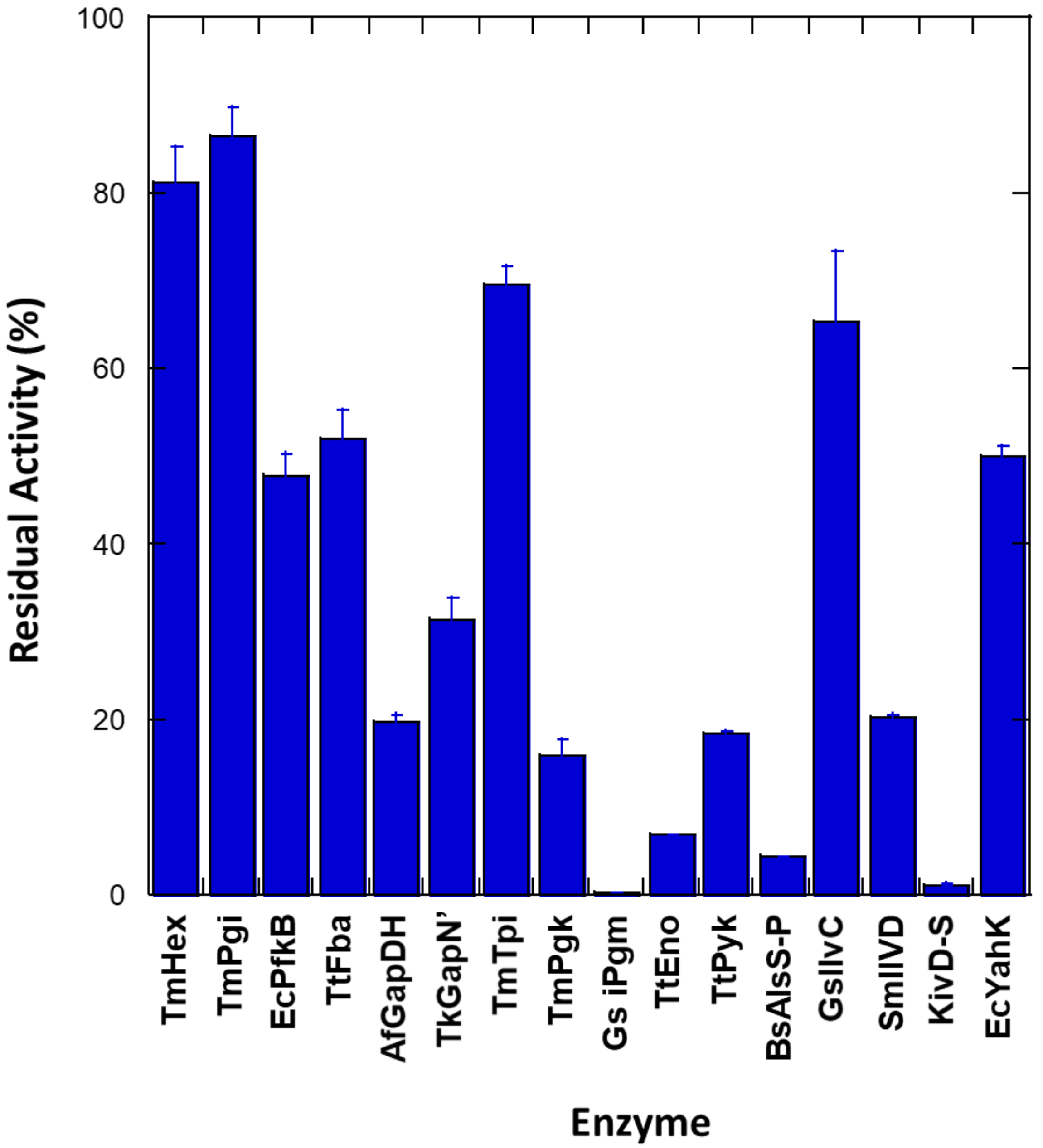

Supplementary Fig. 3. Residual activity of enzymes after 120 hours in bioreactor reaction that included addition of second aliquot of BsAlsS-P and KivD-S. The progress of the reaction is shown in Fig. 5. Error bars represent the mean and standard deviation of triplicates. Source data are provided as a Source Data file.

Supplementary References

1. Kurylenko, O. O., Ruchala, J., Dmytruk, K. V., Abbas, C. A. & Sibirny, A. A. Multinuclear yeast *Magnusiomyces (Dipodascus, Endomyces) magnusii* is a promising isobutanol producer. *Biotechnology Journal*, https://doi.org/10.1002/biot.201900490

2. Park, S.-H. & Hahn, J.-S. Development of an efficient cytosolic isobutanol production pathway in *Saccharomyces cerevisiae* by optimizing copy numbers and expression of the pathway genes based on the toxic effect of α-acetolactate. *Scientific Reports* **9**, 3996 (2019).

3. Zhang, Y. *et al.* Xylose utilization stimulates mitochondrial production of isobutanol and 2-methyl-1-butanol in *Saccharomyces cerevisiae*. *Biotechnology for Biofuels* **12**, 223 (2019).

4. Zhao, E. M. *et al.* Optogenetic regulation of engineered cellular metabolism for microbial chemical production. *Nature* **555**, 683–687 (2018).

5. Lee, W.-H. *et al.* Isobutanol production in engineered *Saccharomyces cerevisiae* by overexpression of 2-ketoisovalerate decarboxylase and valine biosynthetic enzymes. *Bioprocess Biosyst Eng* **35**, 1467–1475 (2012).

6. Avalos, J. L., Fink, G. R. & Stephanopoulos, G. Compartmentalization of metabolic pathways in yeast mitochondria improves the production of branched-chain alcohols. *Nature Biotechnology* **31**, 335–341 (2013).

7. Matsuda, F. *et al.* Increased isobutanol production in *Saccharomyces cerevisiae* by eliminating competing pathways and resolving cofactor imbalance. *Microbial Cell Factories* **12**, 119 (2013).

8. Zhang, Y., Hammer, S. K., Carrasco-Lopez, C., Garcia Echauri, S. A. & Avalos, J. L. Genetically encoded biosensors for branched-chain amino acid metabolism to monitor mitochondrial and cytosolic production of isobutanol and isopentanol in yeast. bioRxiv 2020.03.08.982801 (2020) doi:10.1101/2020.03.08.982801.

9. Promdonkoy, P., Mhuantong, W., Champreda, V., Tanapongpipat, S. & Runguphan, W. Improvement in d-xylose utilization and isobutanol production in *S. cerevisiae* by adaptive laboratory evolution and rational engineering. *J Ind Microbiol Biotechnol* (2020) doi:10.1007/s10295-020-02281-9.

10. Holwerda, E. K. *et al.* Metabolic and evolutionary responses of *Clostridium thermocellum* to genetic interventions aimed at improving ethanol production. *Biotechnology for Biofuels* **13**, 40 (2020).

11. Lin, P. P. *et al.* Consolidated bioprocessing of cellulose to isobutanol using *Clostridium thermocellum*. *Metabolic Engineering* **31**, 44–52 (2015).

12. Higashide, W., Li, Y., Yang, Y. & Liao, J. C. Metabolic engineering of *Clostridium cellulolyticum* for production of isobutanol from cellulose. *Appl. Environ. Microbiol.* **77**, 2727–2733 (2011).

13. Lin, P. P. *et al.* Isobutanol production at elevated temperatures in thermophilic *Geobacillus thermoglucosidasius*. *Metabolic Engineering* **24**, 1–8 (2014).

14. Qi, H. *et al.* Model-driven redox pathway manipulation for improved isobutanol production in *Bacillus subtilis* complemented with experimental validation and metabolic profiling analysis. *PLoS ONE* **9**, e93815 (2014).

15. Choi, K.-Y., Wernick, D. G., Tat, C. A. & Liao, J. C. Consolidated conversion of protein waste into biofuels and ammonia using *Bacillus subtilis*. *Metabolic Engineering* **23**, 53–61 (2014).

16. Boock, J. T. *et al.* Engineered microbial biofuel production and recovery under supercritical carbon dioxide. *Nature Communications* **10**, 587 (2019).

17. Atsumi, S., Higashide, W. & Liao, J. C. Direct photosynthetic recycling of carbon dioxide to isobutyraldehyde. *Nature Biotechnology* **27**, 1177–1180 (2009).

18. Li, H. *et al.* Integrated electromicrobial conversion of CO2 to higher alcohols. *Science* **335**, 1596–1596 (2012).

19. Baez, A., Cho, K.-M. & Liao, J. C. High-flux isobutanol production using engineered Escherichia coli: a bioreactor study with in situ product removal. *Appl. Microbiol. Biotechnol.* **90**, 1681–1690 (2011).

20. Atsumi, S., Hanai, T. & Liao, J. C. Non-fermentative pathways for synthesis of branched-chain higher alcohols as biofuels. *Nature* **451**, 86–89 (2008).

21. Huo, Y.-X. *et al.* Conversion of proteins into biofuels by engineering nitrogen flux. *Nature Biotechnology* **29**, 346–351 (2011).
