# Supplementary material for: Isobutanol production freed from biological limits using synthetic biochemistry
Source: Nat Commun. 2020 Aug 27;11:4292. doi: 10.1038/s41467-020-18124-1 (PMC7453195; doi:10.1038/s41467-020-18124-1)
Supplement: Supplementary file 3 — Description of Additional Supplementary Files [file 41467_2020_18124_MOESM3_ESM.pdf]

**Description of Additional Supplementary Files**

Supplementary Data 1

Protein sequences of enzymes used in this study

Supplementary Data 2

DNA sequences of enzymes used in this study
