# Supplementary material for: Isobutanol production freed from biological limits using synthetic biochemistry
Source: Nat Commun. 2020 Aug 27;11:4292. doi: 10.1038/s41467-020-18124-1 (PMC7453195; doi:10.1038/s41467-020-18124-1)
Supplement: Supplementary file 4 — Supplementary Data 1 [file 41467_2020_18124_MOESM4_ESM.docx]

**Protein sequences of enzymes used in this study**

**(His tag leader sequence is not included)**

**Hexokinase from *Thermotoga maritima* (TmHex)**
NCBI Reference Sequence: WP_010865348

MPKLKLIGVDLGGTTFSVGLVSEDGKILKKVTRDTLVENGKEDVIRRIAETILEVSDGEEAPYVGIGSPG

SIDRENGIVRFSPNFPDWHNVPLTDELAKRTGKKVFLENDANAFVLGEKWFGAGRGHDHIVALTLGTGIG

GGVVTHGYLLTGRDGIGAELGHVVVEPNGPMCNCGTRGCLEAVASATAIRRFLREGYKKYHSSLVYKLAG

SPEKADAKHLFDAARQGDRFALMIRDRVVDALARAVAGYIHIFNPEIVIIGGGISRAGEILFGPLREKVV

DYIMPSFVGTYEVVASPLVEDAGILGAASIIKERIGG

**Glucose-6-phosphate isomerase from *Thermotoga maritima* (TmPgi)**

NCBI Reference Sequence: WP_004081585

MSLKFDFSNLFEPNISGGLTDEDVKSVEEKVTSAVRNFVENTPDFAKLDRSWIDSVKSLEDWIINFDTVV

VLGIGGSGLGNLALHYSLRPLNWNEMTREERNGYARVFVVDNVDPDLMSSVLDRIDPKTTLFNVISKSGS

TAEVMATYSIARGILEAYGLDPREHMLITTDPEKGFLRKLVKEEGFRSLEVPPGVGGRFSVLTPVGLLSA

MAEGIDIDELHEGAKDAFEKSMKENILENPAAMIALTHYLYLNKGKSISVMMAYSNRMIYLVDWYRQLWA

ESLGKRYNLKGEEVFTGQTPVKALGATDQHSQIQLYNEGPNDKVITFLRVENFDREIVIPETGRAELSYL

ARKKLSELLLAEQTGTEEALRENNRPNMRVTFDGLTPYNVGQFFAYYEAATAFMGYLLEINPFDQPGVEL

GKKITFALMGREGYTYEIKERSKKVIIE

**Phosphofructokinase B from *Escherichia coli* (EcPfkB)**

NCBI Accession Number: EFJ64522

MMVRIYTLTLAPSLDSATITPQIYPEGKLRCTAPVFEPGGGGINVARAIAHLGGSATAIFPAGGATGEHL

VSLLADENVPVATVEAKDWTRQNLHVHVEASGEQYRFVMPGAALNEDEFRQLEEQVLEIESGAILVISGS

LPPGVKLEKLTQLISAAQKQGIRCIVDSSGEALSAALAIGNIELVKPNQKELSALVNRELTQPDDVRKAA

QEIVNSGKAKRVVVSLGPQGALGVDSENCIQVVPPPVKSQSTVGAGDSMVGAMTLKLAENASLEEMVRFG

VAAGSAATLNQGTRLCSHDDTQKIYAYLSR

**Fructose-1,6-bisphosphate aldolase from *Thermus thermophiles* (TtFba)**

NCBI Reference Sequence: WP_008633565

MLVTGLEILRKARAEGYGVGAFNTNNMEFTQAILEAAEEMKSPVILALSEGAMKYGGRALTRMVVALAQE

ARVPVAVHLDHGSSYESVLKALREGFTSVMIDKSHEDFETNVRETKRVVEAAHAVGVTVEAELGRLAGIE

EHVAVDEKDALLTNPEEARIFMERTGADYLAVAIGTSHGAYKGKGRPFIDHPRLARIAELVPAPLVLHGA

SAVPQELVERFRAAGGEIGEASGIHPEDIKKAISLGIAKINTDTDLRLAFTALVRETLGKNPKEFDPRKY

LGPAREAVKEVVKSRMELFGSVGRA

**Triosephosphate isomerase from *Thermotoga maritima* (TmTpi)**

NCBI Accession Number: AAA67520

ITRKLILAGNWKMHKTISEAKKFVSLLVNELHDVKEFEIVVCPPFTALSEVGEILSGRNIKLGAQNVFYE

DQGAFTGEISPLMLQEIGVEYVIVGHSERRRIFKEDDEFINRKVKAVLEKGMTPILCVGETLEEREKGLT

FCVVEKQVREGFYGLDKEEAKRVVIAYEPVWAIGTGRVATPQQAQEVHAFIRKLLSEMYDEETAGSIRIL

YGGSIKPDNFLGLIVQKDIDGGLVGGASLKESFIELARIMRGVIS

**Designed Glyceraldehyde-3-phosphate dehydrogenase (non-phosphorylating) from *Thermococcus kodakarensis* (TkGapN’)**

NCBI Reference Sequence: WP_011249656

**-Mutations introduced to enhance specificity

MVEPFVPEGEIFEGIFRQNEGIPEFATYVNGEWVFTGKTAEVRSPIDGSLIARVSLSDMALSNRAVAAAY

SAGRHEIRDTPGEKRLEAFLKVAELIRDSFDDFVTALVLDAGKPLSNARGEVTATIERLEKTTMEFGRLI

GDYIPGDWSAESLGSEGIVKREPYGVVLAISPYNYPLFISTAKIVPALLAGNAVLLKPPTQDPLAPLLLS

RVLQLAGIPESAYHLLTVPGALMDSILADRRIRAVTFTGSTEVGEHILSMGGIKFYHMELGGKDPAVVLD

DAPLEETVEKLVKGMVSYSGQRCDAIRLIIAEEGIYEQLKRELVAALSKIEPENPLEDEDAIMGPLINER

SAEKIEEVYRDALEKGAVPLTGFKRKGAYVWPVLLEASREVLPGLRAFQEDVFGPLTILVKVSNEDEAVE

LANSSRFGLDAAVFSGDDSRARKVARRLEVGAVFINEFPRHGIGYYPFGGMKDSGIGREGIGYSIETLTT

TKTIVRNYRGRGVWDYI

**Glyceraldehyde-3-phosphate dehydrogenase (phosphorylating) from** ***Archaeoglobus fulgidus* (AfGapDH)**

NCBI Reference Sequence: WP_048064727

MKVKVAINGYGTIGKRVADAVSLQDDMEVVGVTKTRPDFEAKLGAKRYPLYVAKPENVELFERAGIEIQG

TIEDLLPKADIVVDCSPNKVGAENKAKYYEKAGIKAIFQGGEKKDVAEVSFNALANYDEAVGKSYVRVVS

CNTTGLTRLIYMLKTNFSIGRIRATMLRRVVDPKEDKKGLVNGIMPDPVAIPSHHGPDVKTVLPDVDIVT

TAFKLPTTLMHVHSLCVEMREAVKAEDVVSALSEEPRIMLISAEDGFTSTAKVIEFARELRLRYDLYENI

VWRESIGVDGNDLFVTQAVHQEAIVVPENIDAIRAMFELAEKEESIRKTNESLGIGKVF

**Phosphoglycerate kinase domain from *Thermotoga maritima* (TmPgk)**
NCBI Reference Sequence: WP_004081072

MEKMTIRDVDLKGKRVIMRVDFNVPVKDGVVQDDTRIRAALPTIKYALEQGAKVILLSHLGRPKGEPSPE

FSLAPVAKRLSELLGKEVKFVPAVVGDEVKKAVEELKEGEVLLLENTRFHPGETKNDPELAKFWASLADI

HVNDAFGTAHRAHASNVGIAQFIPSVAGFLMEKEIKFLSKVTYNPEKPYVVVLGGAKVSDKIGVITNLME

KADRILIGGAMMFTFLKALGKEVGSSRVEEDKIDLAKELLEKAKEKGVEIVLPVDAVIAQKIEPGVEKKV

VRIDDGIPEGWMGLDIGPETIELFKQKLSDAKTVVWNGPMGVFEIDDFAEGTKQVALAIAALTEKGAITV

VGGGDSAAAVNKFGLEDKFSHVSTGGGASLEFLEGKELPGIASIADKKKI

**Phosphoglycerate mutase (2,3-bisphosphoglycerate independent) from *Geobacillus stearothermophilus* (Gs iPgm)**

NCBI Reference Sequence: WP_033015095

MSKKPVALIILDGFALRDETYGNAVAQANKPNFDRYWNEYPHTTLKACGEAVGLPEGQMGNSEVGHLNIG

AGRIVYQSLTRINIAIREGEFDRNETFLAAMNHVKQHGTSLHLFGLLSDGGVHSHIHHLYALLRLAAKEG

VKRVYIHGFLDGRDVGPQTAPQYIKELQEKIKEYGVGEIATLSGRYYSMDRDKRWDRVEKAYRAMVYGEG

PTYRDPLECIEDSYKHGIYDEFVLPSVIVREDGRPVATIQDNDAIIFYNFRPDRAIQISNTFTNEDFREF

DRGPKHPKHLFFVCLTHFSETVAGYVAFKPTNLDNTIGEVLSQHGLRQLRIAETEKYPHVTFFMSGGREE

EFPGEDRILINSPKVPTYDLKPEMSAYEVTDALLKEIEADKYDAIILNYANPDMVGHSGKLEPTIKAVEA

VDECLGKVVDAILAKGGIAIITADHGNADEVLTPDGKPQTAHTTNPVPVIVTKKGIKLRDGGILGDLAPT

MLDLLGLPQPKEMTGKSLIVK

**Phosphoenolpyruvate hydratase/enolase from *Thermus thermophiles* (TtEno)**

NCBI Reference Sequence: WP_011173981

MTTIVGVRAREVLDSRGFPTVEAEVELEGGARGRAMVPSGASTGTHEALELRDGGKRYLGKGVRRAVENV

NERIAPELVGMDALDQEGVDRAMLELDGTPNKANLGANAVLAVSLAVARAAAEALGLPLYRYLGGVQGVT

LPVPLMNVINGGKHADNRVDFQEFMLVPAGAGSFAEALRIGAEVFHTLKAVLKEKGYSTNVGDEGGFAPD

LRSNEEAVELLLLAIERAGYTPGQEVSLALDPATSELYRDGKYHLEGEGKVLSSEEMVAFWEAWVEKYPI

RSIEDGLAEDDWEGWRLLTERLGGKVQLVGDDLFVTNPERLRAGIERGVANAILVKVNQIGTLSETLEAI

RLAQRSGYRAVISHRSGETEDSFIADLAVAVNAGQIKTGSLSRSDRLAKYNQLLRIEEELGRAARFLGYA

AF

**Pyruvate kinase from *Thermus thermophilus* (TtPyk)**

NCBI Reference Sequence: WP_011227631

MPPFKRTKIVATLGPATDDKEVIRALAEAGADVFRLNFSHGAPEDHRRRVGWVREVAEELGRTLAVLQDL

QGPKIRVGRFREGQVLLRPGQRFVLTAEPVEGDEHRVSVSYKGLPEDVSPGQILLLDDGRIRLKVLEVRS

PEILTEVEVGGVLSNNKGINIPGADLSIPALSEKDIQDLALGAELGVDWVAVSFVRTRDDLLLARHYLSR

YGSKARLMAKIEKPSAVARFEEILEEADGIMVARGDLGVEMPLEEVPIVQKRLILRCIAAGKPVITATQM

LESMVQNPSPTRAEASDVANAIFDGTDAVMLSAETAAGAYPVEAVAMMARIAKAVESSPEFLQKLNVLRP

APTPTTQDAIAQAADDVVEAVGARAIVVFTATGGSARRIARTRPQVPILALTPNPEVRNQLALVWGVYPH

LAPDPQDTDDMVRIALREVKALGLAQVGDRVVIAAGVPFGVRGTTNLIRVERVG

**Designed Acetolactate synthase (BsAlsS-P)**

*this study

**-Mutations introduced to increase stability

MLTKATKEQKSLVKNRGAELVVDCLINQGVTHVFGIPGAKIDAVFDALQDKGPEIIVARHEQNAAFMAQAVGRLTGKPGVVLVTSGPGASNLATGLLTANTEGDPVVALAGNVPRADRLKRTHQSLDNAALFQPITKYSVEVQDPDNIPEAVTNAFRIAEAGQAGAAFVSFPQDVVNEVTNTKNVRPVAPPKLGPAPDDAISAAIAKIQTAKLPVVLVGMKGGRPEAIKAIRKLLKKTQLPFVETYQAAGTLSRDLEDLYFGRIGLFRNQPGDLLLEQADVVLTIGYDPIEYDPKFWNINGDRTIIHLDEIPADIDHYYQPDLELIGDIPSTINHIAHDAVKVEFSEREQKILSDLKQYMHELEQVPADWKSDRAHPLEIVKELRNAVDDDVTVTCDIGSHAIWMARYFRSYEPLTLMISNGMQTLGVALPWAIGASLVKPGEKVVSVSGDGGFLFSAMELETAVRLKAPIVHIVWNDSTYDMVAFQQLKKYNRTSGVDFGNIDIVKYAESFGATGLRVESPDQLADVLRQGMNAEGPVIIDVPVDYSDNINLASDKLPKEFGELMKTKAL

**Ketol-acid reductoisomerase from *Geobacillus stearothermophilus* (GsIlvC)**

NCBI Reference Sequence: WP_033014337

MAKVYYNGDANEQYLQGKTVAIIGYGSQGHAHAQNLRDSGVRVIVGLRKGKSWEQAEQDGFEVYSVREAA

KQADIVMVLLPDEKQPAVYKEEIEPGLEPGNALVFAHGFNIHFSQIVPPEHVDVFLVAPKGPGHLVRRTY

AEGAGVPALIAVYQDVTGHAKETALAYAKAIGAARAGVLETTFKEETETDLFGEQAVLCGGLTALIKAGF

ETLVEAGYQPEVAYFECLHEMKLIVDLLYEGGLSWMRYSISDTAQWGDFITGPRIINDAVKAEMKKVLDD

IQTGKFAKSWILENQANRPEFNAINRRENEHLIEVVGRELRSMMPFVKAKQKEAVVPGAKH

**Dihydroxyacid dehydratase from *Streptococcus mutans* (SmIlvD)**

NCBI Accession Number: ESS16062

MMKGSGQMTDKKTLKDLRNRSSVYDSMVKSPNRAMLRATGMQDEDFEKPIVGVISTWAENTPCNIHLHDF

GKLAKVGVKEAGAWPVQFGTITVSDGIAMGTQGMRFSLTSRDIIADSIEAAMGGHNADAFVAIGGCDKNM

PGSVIAMANMDIPAIFAYGGTIAPGNLDGKDIDLVSVFEGVGHWNHGDMTKEEVKALECNACPGPGGCGG

MYTANTMATAIEVLGLSLPGSSSHPAESAEKKADIEEAGRAVVKMLEMGLKPSDILTREAFEDAITVTMA

LGGSTNSTLHLLAIAHAANVELTLDDFNTFQEKVPHLADLKPSGQYVFQDLYKVGGVPAVMKYLLKNGFL

HGDRITCTGKTVAENLKAFDDLTPGQKVIMPLENPKREDGPLIILHGNLAPDGAVAKVSGVKVRRHVGPA

KVFNSEEEAIEAVLNDDIVDGDVVVVRFVGPKGGPGMPEMLSLSSMIVGKGQGEKVALLTDGRFSGGTYG

LVVGHIAPEAQDGGPIAYLQTGDIVTIDQDTKELHFDISDEELKHRQETIELPPLYSRGVLGKYAHIVSS

ASRGAVTDFWKPEETGKK

**Aldehyde reductase from *Escherichia coli* (EcYahK)**

NCBI Reference Sequence: WP_128424329

MKIKAVGAYSAKQPLEPMDITRREPGPNDVKIEIAYCGVCHSDLHQVRSEWAGTVYPCVPGHEIVGRVVA

VGDQVEKYAPGDLVGVGCIVDSCKHCEECEDGLENYCDHMTGTYNSPTPDEPGHTLGGYSQQIVVHERYI

LRIRHPQEQLAAVAPLLCAGITTYSPLRHWQAGPGKKVGVVGIGGLGHMGIKLAHAMGAHVVAFTTSEAK

REAAKALGADEVVNSRNADEMAAHLKSFDFILNTVAAPHNLDDFTTLLKRDGTMTLVGAPATPHKSPEVF

NLIMKRRAIAGSMIGGIPETQEMLDFCAEHGIVADIEMIRADQINEAYERMLRGDVKYRFVIDNRTLTD

**KivD-S Protein Sequence**

**-Leader sequence

**-LLM3 Mutations

**-Mutations suggested by the PROSS algorithm

**- PROSS mutants that we reverted to wild-type due to slow inactivation phenomenon

**-Mutations introduced by directed evolution

MGSS**HHHHHH**SSG**LVPRGS**HMYTVGDYLLDRLHELGIEHIFGVPGDYNLQFLDHIISRKDMKWVGNANELNASYMADGYARTK**K**AAAFLTTFGVGELSAVNGLAGSYAENLPVV**E**IVGSPTSKVQNEGKFVHHTLADGDFKHFMKMHEPVTAARTLLTAENATVEIDRVLSALLKERKPVYINLPVDVAAAKAEKPSLPLKKENSTSNTSDQEILNKIQESLKNAKKPIVITGHEIISFGLEKTVSQFISKTKLPITTLNFGK**S**SVDESLPSFLGIYNGKLSEPNLKEFVESADFILMLGVKLTDSSTGVFTHHLDENKMISLNIDEAKIFGESIQNFDFESLIKSLLDLSEIEYKGKYIDKKQEDFVPSNALLSQDRLWQAVEHLTQSNEVIVAEQGTSFFGVSPIRLKPGSTFIGQPLWGSIGYTFPAALGSQIADPERRHILFIGDGSLQMSVQELGLAIREGINPIIFIINNDGYTVEREIHGPNQSYNDIPMWNYSKLPESFGATEERVVSKIVTTENEFVSVMKEAQADPNRMYWIELILAKEDAPKVLKKMGKLLAEQNK
