# Supplementary material for: Isobutanol production freed from biological limits using synthetic biochemistry
Source: Nat Commun. 2020 Aug 27;11:4292. doi: 10.1038/s41467-020-18124-1 (PMC7453195; doi:10.1038/s41467-020-18124-1)
Supplement: Supplementary file 5 — Supplementary Data 2 [file 41467_2020_18124_MOESM5_ESM.docx]

**DNA sequences of enzymes used in this study**

**DNA** – His tag coding sequence

**Hexokinase from *Thermotoga maritima* (TmHex)**
ATGGGCAGCAGCCATCATCATCATCATCACAGCAGCGGCCTGGTGCCGCGCGGCAGCCATATG

CCGAAGTTGAAGCTCATAGGAGTTGATCTTGGAGGAACTACCTTCTCGGTAGGACTCGTGAGTGAAG

ATGGGAAAATACTGAAGAAAGTAACTCGAGATACTCTCGTAGAGAACGGAAAAGAAGATGTGATAAGAAG

AATCGCTGAGACGATCCTGGAGGTATCTGACGGGGAAGAAGCGCCCTATGTTGGTATAGGATCTCCAGGT

TCGATAGATAGAGAGAATGGGATCGTCAGATTTTCTCCAAATTTTCCGGACTGGCACAACGTTCCACTAA

CCGATGAACTGGCAAAGAGAACCGGGAAAAAGGTTTTTCTTGAAAACGATGCGAACGCGTTTGTTCTTGG

AGAGAAGTGGTTCGGCGCTGGCAGAGGGCATGATCACATAGTGGCTCTGACACTTGGAACAGGTATTGGC

GGGGGAGTTGTCACCCATGGATACCTCCTCACGGGAAGGGATGGAATAGGAGCAGAACTCGGGCATGTTG

TTGTTGAACCCAATGGCCCCATGTGTAACTGTGGCACGAGAGGTTGTCTTGAAGCTGTGGCATCCGCAAC

GGCGATAAGGAGATTTCTCAGAGAGGGCTACAAGAAATATCACAGCTCTCTGGTGTACAAACTCGCAGGT

TCACCTGAAAAGGCAGATGCCAAGCACCTCTTCGACGCCGCAAGACAGGGAGACAGATTCGCTCTGATGA

TAAGAGACAGGGTGGTGGACGCCCTGGCACGAGCCGTAGCGGGCTACATACACATATTCAACCCGGAGAT

AGTGATCATAGGTGGAGGAATTTCAAGAGCGGGAGAGATTCTGTTTGGTCCCCTGAGAGAGAAAGTGGTG

GACTACATCATGCCATCCTTTGTGGGAACCTACGAAGTAGTGGCGAGTCCTCTTGTCGAAGACGCCGGAA

TCCTTGGGGCGGCTTCCATCATAAAGGAGAGGATAGGGGGGTaA

**Glucose-6-phosphate isomerase from *Thermotoga maritima* (TmPgi)**

ATGGGCAGCAGCCATCATCATCATCATCACAGCAGCGGCCTGGTGCCGCGCGGCAGCCATATG

AGTTTAAAATTCGACTTTTCCAATTTGTTTGAACCGAACATCTCCGGTGGTTTGACAGATGAAGATG

TAAAAAGTGTAGAGGAGAAAGTTACAAGTGCAGTTCGAAACTTTGTAGAAAACACTCCCGATTTTGCAAA

GCTGGACAGAAGCTGGATCGATTCTGTTAAATCTCTCGAGGACTGGATAATAAACTTCGATACGGTCGTT

GTGCTGGGAATTGGAGGTTCTGGACTTGGAAACCTGGCCCTTCACTACTCCCTCAGGCCTTTGAACTGGA

ACGAGATGACCAGAGAGGAGAGAAACGGATACGCCAGAGTGTTTGTGGTGGACAACGTAGATCCAGATCT

CATGAGCTCTGTTCTGGACAGAATCGATCCCAAAACAACTCTTTTCAACGTGATATCGAAATCCGGTTCC

ACCGCTGAAGTAATGGCGACCTATTCGATAGCACGTGGGATCCTCGAAGCTTATGGACTAGATCCAAGAG

AACACATGCTCATCACAACAGACCCGGAAAAGGGCTTTTTGAGAAAACTCGTGAAAGAAGAAGGTTTCAG

AAGTCTCGAAGTGCCTCCTGGAGTGGGAGGAAGGTTCAGCGTTCTCACACCTGTTGGCCTCCTCTCAGCC

ATGGCGGAAGGCATAGACATAGACGAACTGCACGAAGGTGCAAAGGATGCCTTCGAGAAGAGCATGAAAG

AGAACATCCTTGAAAATCCAGCGGCAATGATAGCACTCACACACTACCTCTACTTGAATAAAGGAAAGTC

CATTTCTGTGATGATGGCGTATTCGAACAGAATGATCTATCTTGTGGACTGGTACAGACAGCTCTGGGCG

GAAAGTCTTGGAAAAAGATACAATCTCAAGGGAGAAGAGGTTTTCACCGGTCAAACTCCTGTGAAAGCGC

TGGGAGCGACCGATCAGCACTCACAGATACAGCTTTACAACGAAGGGCCGAACGACAAGGTAATAACCTT

CCTGAGAGTTGAAAACTTCGACAGAGAAATAGTGATCCCGGAAACCGGAAGAGCTGAACTCAGCTATCTT

GCAAGGAAGAAGCTCTCCGAACTCCTCCTCGCAGAGCAGACAGGAACAGAAGAAGCACTGCGAGAAAACA

ACAGACCGAACATGAGAGTGACATTTGACGGACTCACACCGTACAACGTGGGCCAATTCTTCGCTTACTA

CGAGGCTGCCACCGCTTTCATGGGTTATCTTCTTGAGATCAACCCGTTCGATCAGCCCGGTGTGGAGCTC

GGAAAAAAGATCACGTTCGCTCTTATGGGAAGAGAAGGTTACACTTACGAAATAAAGGAAAGGTCGAAGA

AGGTGATTATAGAATAA

**Phosphofructokinase B from *Escherichia coli* (EcPfkB)**

ATGGGCAGCAGCCATCATCATCATCATCACAGCAGCGGCCTGGTGCCGCGCGGCAGCCATATG

GTACGTATCTATACGTTGACACTTGCGCCCTCTCTCGATAGCGCAACAATTACCCCGCAAATTTATC

CCGAAGGAAAACTGCGCTGTACCGCACCGGTGTTCGAACCCGGGGGCGGCGGCATCAACGTCGCCCGCGC

CATTGCCCATCTTGGAGGCAGTGCCACAGCGATCTTCCCGGCGGGTGGCGCGACCGGCGAACACCTGGTT

TCACTGTTGGCGGATGAAAATGTCCCCGTCGCTACTGTAGAAGCCAAAGACTGGACCCGGCAGAATTTAC

ACGTACATGTGGAAGCAAGCGGTGAGCAGTATCGTTTTGTTATGCCAGGCGCGGCATTAAATGAAGATGA

GTTTCGCCAGCTTGAAGAGCAAGTTCTGGAAATTGAATCCGGGGCCATCCTGGTCATAAGCGGAAGCCTG

CCGCCAGGTGTGAAGCTGGAAAAATTAACCCAACTGATTTCCGCTGCGCAAAAACAAGGGATCCGCTGCA

TCGTCGACAGTTCTGGCGAAGCGTTAAGTGCAGCACTGGCAATTGGTAACATCGAGTTGGTTAAGCCTAA

CCAAAAAGAACTCAGTGCGCTGGTGAATCGCGAACTCACCCAGCCGGACGATGTCCGCAAAGCCGCGCAG

GAAATCGTTAATAGCGGCAAGGCCAAACGGGTTGTCGTTTCCCTGGGTCCACAAGGAGCGCTGGGTGTTG

ATAGTGAAAACTGTATTCAGGTGGTGCCACCACCGGTGAAAAGCCAGAGTACCGTTGGCGCTGGTGACAG

CATGGTCGGCGCGATGACACTGAAACTGGCAGAAAATGCCTCTCTTGAAGAGATGGTTCGTTTTGGCGTA

GCTGCGGGGAGTGCAGCCACACTCAATCAGGGAACACGTCTGTGCTCCCATGACGATACGCAAAAAATTT

ACGCTTACCTTTCCCGCTAA

**Fructose-1,6-bisphosphate aldolase from *Thermus thermophiles* (TtFba)**

ATGGGCAGCAGCCATCATCATCATCATCACAGCAGCGGCCTGGTGCCGCGCGGCAGCCATATG

CTGGTAACGGGTCTAGAGATCTTGCGCAAGGCGCGGGCGGAAGGCTACGGGGTCGGGGCCTTCAACA

CCAACAACATGGAGTTCACCCAGGCCATCCTCGAGGCCGCCGAGGAAATGAAAAGCCCCGTGATCCTCGC

CCTCTCCGAGGGGGCGATGAAGTACGGGGGCCGGGCCCTCACCCGCATGGTGGTGGCCCTGGCCCAGGAG

GCCCGGGTGCCCGTGGCCGTGCACCTGGACCACGGCTCCAGCTACGAGAGCGTCCTCAAGGCCCTCAGGG

AGGGCTTCACCAGCGTCATGATTGACAAGTCCCACGAGGACTTTGAGACGAACGTCCGGGAGACCAAGCG

GGTGGTGGAGGCGGCCCACGCCGTGGGGGTCACGGTGGAGGCGGAGCTCGGGCGGCTTGCGGGGATTGAG

GAGCACGTGGCCGTGGACGAGAAGGACGCCCTCCTCACCAACCCCGAGGAGGCCCGGATCTTCATGGAGC

GCACGGGGGCCGACTACTTGGCGGTGGCCATCGGCACGAGCCACGGGGCCTACAAGGGGAAGGGGAGGCC

CTTCATTGACCACCCCCGGCTCGCCCGCATCGCCGAGCTCGTCCCTGCCCCCCTCGTCCTCCACGGGGCG

AGCGCCGTGCCCCAAGAGCTCGTGGAGCGCTTCCGGGCCGCAGGGGGCGAGATCGGGGAGGCCTCGGGCA

TCCACCCCGAGGACATCAAGAAGGCCATCTCCTTGGGCATCGCCAAGATCAACACCGACACCGACCTGCG

CCTCGCCTTCACCGCTCTTGTCCGGGAGACCCTGGGGAAAAACCCCAAGGAGTTTGACCCCAGGAAGTAC

CTGGGCCCCGCCCGGGAGGCGGTGAAGGAGGTGGTGAAAAGCCGCATGGAGCTTTTCGGCTCCGTGGGGC

GGGCTTAG

**Triosephosphate isomerase from *Thermotoga maritima* (TmTpi)**

ATGGGCAGCAGCCATCATCATCATCATCACAGCAGCGGCCTGGTGCCGCGCGGCAGCCATATG

ATAACTCGTAAACTGATCCTCGCTGGGAACTGGAAGATGCATAAAACGATCTCGGAAGCGAAAAAGTTTG

TGTCGCTGCTCGTGAACGAACTTCACGACGTGAAAGAGTTCGAAATAGTGGTCTGTCCTCCGTTCACAGC

TCTATCTGAAGTGGGGGAAATACTCTCTGGTAGAAACATCAAATTGGGAGCTCAAAACGTTTTCTACGAA

GACCAGGGAGCGTTCACCGGGGAGATTTCTCCTCTCATGCTGCAAGAGATCGGCGTTGAATACGTGATCG

TGGGACATTCCGAGAGAAGGCGTATTTTCAAAGAAGACGACGAGTTCATAAACAGGAAAGTGAAAGCGGT

GCTTGAAAAAGGTATGACTCCTATTCTCTGCGTTGGAGAAACACTCGAGGAAAGAGAGAAAGGGCTCACT

TTCTGCGTTGTGGAAAAACAGGTGAGAGAAGGTTTCTACGGTCTCGACAAAGAGGAAGCAAAGAGAGTGG

TAATAGCTTACGAGCCAGTCTGGGCAATCGGGACAGGAAGGGTGGCGACACCACAGCAGGCACAGGAAGT

ACACGCGTTCATAAGAAAGCTGCTCTCAGAGATGTACGACGAGGAAACAGCGGGATCGATAAGGATTCTC

TACGGTGGAAGCATAAAGCCGGACAATTTCCTCGGTCTCATCGTTCAGAAGGATATAGATGGTGGTCTCG

TTGGAGGAGCGAGTCTCAAAGAGTCTTTCATAGAACTTGCACGAATAATGAGAGGTGTGATTTCCTAA

**Designed Glyceraldehyde-3-phosphate dehydrogenase (non-phosphorylating) from *Thermococcus kodakarensis* (TkGapN’)**

**DNA** – Mutations introduced

ATGGGCAGCAGCCATCATCATCATCATCACAGCAGCGGCCTGGTGCCGCGCGGCAGCCATATG

GTTGAACCGTTCGTTCCGGAAGGTGAAATCTTCGAAGGTATCTTCCGTCAGAACGAAGGTATCCCGGAATTCGCGACCTACGTTAACGGTGAATGGGTTTTCACCGGTAAAACCGCGGAAGTTCGTTCTCCGATCGACGGTTCTCTGATCGCGCGTGTTTCTCTGTCTGACATGGCGCTGTCTAACCGTGCGGTTGCGGCGGCGTACTCTGCGGGTCGTCACGAAATCCGTGACACCCCGGGTGAAAAACGTCTGGAAGCGTTCCTGAAAGTTGCGGAACTGATCCGTGACTCTTTCGACGACTTCGTTACCGCGCTGGTTCTGGACGCGGGTAAACCGCTGTCTAACGCGCGTGGTGAAGTTACCGCGACCATCGAACGTCTGGAAAAAACCACCATGGAATTCGGTCGTCTGATCGGTGACTACATCCCGGGTGACTGGTCTGCGGAATCTCTGGGTTCTGAAGGTATCGTTAAACGTGAACCGTACGGTGTTGTTCTGGCGATCTCTCCGTACAACTACCCGCTGTTCATCTCTACCGCGAAAATCGTTCCGGCGCTGCTGGCGGGTAACGCGGTTCTGCTGAAACCGCCGACCCAGGACCCGCTGGCGCCGCTGCTGCTGTCTCGTGTTCTGCAGCTGGCGGGTATCCCGGAATCTGCGTACCACCTGCTGACCGTTCCGGGTGCGCTGATGGACTCTATCCTGGCGGACCGTCGTATCCGTGCGGTTACCTTCACCGGTTCTACCGAAGTTGGTGAACACATCCTGTCTATGGGTGGTATCAAATTCTACCACATGGAACTGGGTGGTAAAGACCCGGCGGTTGTTCTGGACGACGCGCCGCTGGAAGAAACCGTTGAAAAACTGGTTAAAGGTATGGTTTCTTACTCTGGTCAGCGTTGCGACGCGATCCGTCTGATCATCGCGGAAGAAGGTATCTACGAACAGCTGAAACGTGAACTGGTTGCGGCGCTGTCTAAAATCGAACCGGAAAACCCGCTGGAAGACGAAGACGCGATCATGGGTCCGCTGATCAACGAACGTTCTGCGGAAAAAATCGAAGAAGTTTACCGTGACGCGCTGGAAAAAGGTGCGGTTCCGCTGACCGGTTTCAAACGTAAAGGTGCGTACGTTTGGCCGGTTCTGCTGGAAGCGTCTCGTGAAGTTCTGCCGGGTCTGCGTGCGTTCCAGGAAGACGTTTTCGGTCCGCTGACCATCCTGGTTAAAGTTTCTAACGAAGACGAAGCGGTTGAACTGGCGAACTCTTCTCGTTTCGGTCTGGACGCGGCGGTTTTCTCTGGTGACGACTCTCGTGCGCGTAAAGTTGCGCGTCGTCTGGAAGTTGGTGCGGTTTTCATCAACGAATTCCCGCGTCACGGTATCGGTTACTACCCGTTCGGTGGTATGAAAGACTCTGGTATCGGTCGTGAAGGTATCGGTTACTCTATCGAAACCCTGACCACCACCAAAACCATCGTTCGTAACTACCGTGGTCGTGGTGTTTGGGACTACATCTAA

**Glyceraldehyde-3-phosphate dehydrogenase (phosphorylating) from** ***Archaeoglobus fulgidus* (AfGapDH)**

ATGGGCAGCAGCCATCATCATCATCATCACAGCAGCGGCCTGGTGCCGCGCGGCAGCCATATG

AAGGTTAAGGTGGCGATAAACGGTTACGGAACCATCGGAAAGAGAGTGGCTGATGCAGTCAGCCTTC

AGGACGATATGGAGGTTGTCGGAGTTACCAAAACCCGCCCAGACTTTGAGGCGAAACTCGGCGCCAAAAG

GTATCCTCTCTACGTGGCCAAGCCCGAGAACGTGGAGCTTTTCGAGAGGGCGGGGATCGAGATTCAGGGA

ACAATAGAGGATTTGCTGCCAAAAGCCGACATCGTTGTCGACTGCAGCCCGAACAAGGTTGGGGCGGAAA

ACAAGGCGAAGTATTACGAGAAAGCGGGCATAAAGGCGATATTCCAGGGCGGGGAGAAGAAGGACGTTGC

CGAGGTCTCCTTCAACGCGCTGGCCAACTATGATGAGGCTGTTGGGAAAAGCTACGTAAGGGTTGTGAGC

TGTAACACCACCGGTTTGACGAGGCTCATTTACATGCTCAAAACGAACTTCAGCATCGGCAGGATTAGGG

CGACGATGCTGAGAAGGGTTGTCGACCCGAAGGAAGACAAGAAGGGGCTCGTCAACGGAATCATGCCTGA

TCCGGTTGCGATTCCATCCCACCACGGGCCGGACGTCAAGACTGTTTTGCCCGATGTTGATATTGTAACA

ACAGCATTCAAGCTCCCCACAACGCTTATGCACGTCCACTCCCTCTGCGTTGAGATGAGAGAAGCGGTCA

AAGCAGAAGACGTCGTGAGCGCTCTCAGCGAGGAGCCGAGAATCATGCTTATTTCCGCAGAGGATGGCTT

CACCTCAACGGCAAAGGTAATCGAGTTCGCGAGAGAGCTCAGGCTCAGGTACGACCTCTACGAGAACATC

GTTTGGAGGGAATCGATCGGCGTTGATGGAAACGACCTCTTCGTAACGCAGGCGGTGCATCAGGAGGCTA

TTGTTGTTCCGGAGAACATTGACGCCATAAGGGCCATGTTCGAGCTTGCGGAGAAGGAGGAGAGCATAAG

GAAGACGAACGAGAGCCTTGGAATTGGGAAAGTTTTTTAA

**Phosphoglycerate kinase domain from *Thermotoga maritima* (TmPgk)**
ATGGGCAGCAGCCATCATCATCATCATCACAGCAGCGGCCTGGTGCCGCGCGGCAGCCATATG

GAAAAAATGACCATAAGAGATGTTGATCTGAAAGGCAAGAGAGTCATAATGAGAGTGGACTTCAACG

TTCCAGTGAAGGATGGGGTCGTTCAGGACGACACGAGAATAAGAGCCGCCCTTCCAACGATAAAGTACGC

TCTTGAACAGGGTGCAAAGGTGATACTCCTGTCCCATCTTGGAAGGCCCAAGGGAGAACCTTCACCAGAG

TTCAGCCTCGCACCTGTCGCCAAAAGACTCTCTGAGCTTCTTGGAAAAGAAGTGAAGTTTGTTCCCGCTG

TTGTCGGTGATGAGGTGAAAAAGGCCGTTGAGGAGCTCAAAGAGGGAGAGGTTCTCCTCCTTGAAAACAC

CAGATTCCACCCGGGAGAGACCAAGAACGATCCTGAACTCGCGAAGTTCTGGGCTAGCCTCGCCGATATT

CACGTGAACGATGCCTTCGGAACGGCGCACAGGGCACACGCTTCCAACGTTGGAATCGCACAGTTTATTC

CCAGCGTAGCGGGATTCCTCATGGAAAAAGAGATAAAGTTCCTTTCCAAGGTGACTTATAATCCAGAAAA

ACCGTACGTTGTGGTTCTTGGAGGAGCAAAGGTATCTGACAAAATCGGCGTCATCACGAACCTCATGGAG

AAAGCCGACAGAATTCTCATAGGTGGAGCCATGATGTTCACCTTCCTGAAGGCTCTTGGCAAAGAGGTTG

GATCGTCCAGGGTTGAAGAAGACAAGATCGACCTCGCAAAAGAACTCCTCGAAAAAGCGAAAGAAAAGGG

TGTTGAGATCGTTCTTCCCGTTGATGCCGTTATCGCTCAGAAGATCGAACCCGGTGTGGAAAAGAAGGTT

GTCAGAATCGACGACGGGATACCCGAAGGATGGATGGGCCTCGACATAGGACCCGAGACAATTGAGCTCT

TCAAGCAGAAGCTCTCCGATGCAAAAACCGTTGTCTGGAACGGGCCAATGGGAGTCTTCGAAATAGACGA

TTTCGCTGAAGGCACGAAGCAGGTCGCACTTGCGATCGCAGCGCTCACGGAAAAGGGAGCGATCACCGTT

GTGGGTGGAGGAGACAGCGCCGCGGCGGTGAACAAGTTCGGTCTGGAAGACAAATTCTCCCACGTTTCAA

CGGGCGGAGGGGCTTCTCTCGAATTCCTTGAAGGAAAAGAACTTCCTGGTATTGCCAGCATCGCGGATAA

AAAAAAAATATAA

**Phosphoglycerate mutase (2,3-bisphosphoglycerate independent) from *Geobacillus stearothermophilus* (Gs iPgm)**

ATGGGCAGCAGCCATCATCATCATCATCACAGCAGCGGCCTGGTGCCGCGCGGCAGCCATATG

AGTAAAAAACCGGTTGCGCTCATCATTTTAGACGGATTTGCGCTGCGCGACGAAACGTACGGCAATGCGGTCGCTCAGGCGAACAAACCGAACTTTGACCGCTATTGGAACGAATACCCGCACACAACGCTCAAGGCGTGCGGCGAGGCGGTCGGGCTTCCGGAAGGGCAGATGGGCAACTCGGAAGTCGGCCATCTCAACATCGGCGCCGGGCGCATTGTGTACCAAAGCTTAACGCGAGTCAACATTGCCATTCGCGAAGGCGAGTTTGACCGAAATGAAACGTTTTTGGCGGCGATGAACCATGTGAAACAACATGGGACAAGCTTGCATTTGTTCGGCTTGCTTTCCGACGGCGGGGTGCACAGCCATATTCACCATTTGTACGCCCTCTTGCGCTTGGCGGCGAAAGAAGGCGTAAAACGCGTGTACATCCACGGCTTTTTGGACGGCCGCGACGTCGGCCCACAAACAGCGCCGCAATACATCAAAGAACTGCAGGAAAAAATCAAGGAATATGGCGTCGGCGAAATCGCGACGTTATCGGGCCGCTACTACTCGATGGACCGCGACAAGCGGTGGGACCGCGTCGAAAAGGCGTATCGGGCGATGGTGTACGGGGAAGGGCCGACGTACCGCGATCCGCTCGAATGCATCGAGGACTCGTACAAACACGGCATTTACGACGAATTCGTCCTGCCGTCGGTCATCGTCCGCGAAGACGGCCGGCCGGTGGCGACGATTCAAGACAATGACGCGATTATCTTCTATAATTTCCGCCCTGACCGGGCGATCCAAATTTCAAACACGTTTACGAACGAAGATTTCCGCGAGTTTGACCGCGGCCCGAAACATCCGAAGCATTTGTTCTTTGTCTGCTTGACCCATTTCAGCGAAACGGTGAAAGGGTACGTGGCGTTCAAGCCGACGAACCTTGACAACACGCTTGGGGAAGTGCTGTCGCAGCACGGACTGCGCCAACTGCGCATCGCCGAGACCGAAAAATATCCGCACGTGACGTTTTTTATGAGCGGCGGCCGCGAAGAGAAATTTCCAGGCGAAGACCGGATTTTGATCAACTCGCCGAAAGTGCCGACGTATGACTTGAAGCCGGAAATGAGCGCCTATGAAGTGACCGATGCGCTGCTCAAGGAAATTGAAGCCGATAAGTACGATGCGATCATTTTGAACTACGCCAACCCGGATATGGTCGGCCATTCGGGCAAGCTCGAACCGACGATCAAGGCGGTGGAGGCAGTGGACGAATGCCTCGGCAAAGTCGTCGATGCCATTTTGGCCAAAGGCGGCATCGCCATCATCACCGCCGACCACGGCAACGCCGATGAAGTATTGACGCCGGACGGCAAGCCGCAAACGGCTCATACGACGAATCCGGTGCCGGTCATCGTGACGAAAAAAGGCATCAAGCTTAGAGACGGCGGCATCTTAGGCGATTTGGCGCCGACGATGCTCGATTTGCTCGGCTTGCCGCAGCCGAAAGAAATGACGGGGAAATCGTTGATTGTCAAATAA

**Phosphoenolpyruvate hydratase/enolase from *Thermus thermophiles* (TtEno)**

ATGGGCAGCAGCCATCATCATCATCATCACAGCAGCGGCCTGGTGCCGCGCGGCAGCCATATG

ACCACCATCGTCGGCGTCCGGGCACGCGAGGTTTTGGATTCCAGGGGCTTTCCCACGGTAGAGGCGG

AGGTGGAGCTGGAAGGCGGGGCCAGGGGCCGGGCCATGGTGCCCTCCGGGGCCTCCACCGGAACCCACGA

GGCCCTGGAGCTCAGGGACGGCGGCAAGCGCTACCTGGGCAAGGGGGTGCGCCGGGCGGTGGAGAACGTC

AACGAGCGCATCGCCCCCGAGCTCGTCGGCATGGACGCCCTGGACCAGGAAGGGGTGGACCGGGCCATGC

TGGAGCTGGACGGCACCCCCAACAAGGCCAACCTGGGGGCGAACGCCGTCCTCGCGGTCTCCCTGGCCGT

GGCCCGGGCGGCGGCCGAGGCCCTGGGCCTGCCCCTTTACCGCTACCTGGGCGGGGTCCAGGGGGTCACC

CTGCCCGTGCCCCTCATGAACGTCATCAACGGGGGGAAGCACGCCGACAACCGGGTGGACTTCCAGGAGT

TCATGCTGGTGCCCGCGGGGGCGGGAAGCTTCGCCGAGGCCTTGAGGATCGGGGCCGAGGTCTTCCACAC

CCTCAAGGCCGTCCTCAAGGAGAAGGGCTACAGCACCAACGTGGGGGACGAGGGGGGCTTCGCCCCCGAC

CTCAGGAGCAACGAGGAGGCGGTGGAGCTTTTGCTCCTCGCCATTGAGCGGGCGGGGTACACCCCGGGCC

AGGAGGTCTCCCTGGCCCTGGACCCGGCCACGAGCGAGCTTTACCGGGACGGGAAGTACCACCTGGAGGG

GGAGGGCAAGGTCCTCTCCTCGGAGGAGATGGTGGCCTTCTGGGAGGCCTGGGTGGAGAAGTACCCCATC

CGCTCCATTGAGGACGGCCTCGCCGAGGACGACTGGGAGGGGTGGCGGCTTCTCACCGAGCGCCTGGGGG

GGAAGGTCCAGCTCGTGGGGGACGACCTCTTCGTCACCAACCCGGAAAGGCTCCGGGCGGGGATTGAGCG

GGGGGTGGCCAACGCCATCCTGGTCAAGGTGAACCAGATCGGGACCCTCTCGGAGACCCTCGAGGCCATC

CGCCTGGCCCAGCGCTCGGGGTACAGGGCGGTGATCAGCCACCGCTCCGGGGAGACGGAGGACAGCTTCA

TCGCCGACCTCGCCGTGGCGGTGAACGCCGGACAGATCAAGACCGGTTCCCTTTCCCGCTCCGACCGGCT

GGCCAAGTACAACCAGCTCCTGCGCATTGAGGAGGAGCTGGGCCGGGCCGCGAGGTTTTTGGGGTATGCC

GCCTTTTAA

**Pyruvate kinase from *Thermus thermophilus* (TtPyk)**

ATGGGCAGCAGCCATCATCATCATCATCACAGCAGCGGCCTGGTGCCGCGCGGCAGCCATATG

CCGCCTTTTAAGCGCACCAAGATCGTGGCCACCCTGGGGCCGGCCACGGACGACAAGGAGGTGATCC

GCGCCCTGGCGGAGGCCGGGGCCGACGTCTTCCGCCTGAACTTCAGCCACGGCGCCCCCGAGGACCACAG

GCGCCGGGTGGGCTGGGTGCGGGAGGTGGCGGAGGAGCTCGGCCGAACCCTGGCCGTCCTCCAGGACCTC

CAGGGCCCGAAGATCCGTGTGGGCCGTTTCCGGGAGGGCCAGGTTCTCCTCCGCCCGGGGCAGAGGTTCG

TCCTCACCGCCGAGCCCGTGGAGGGGGACGAGCACCGCGTTTCCGTGAGCTACAAGGGGCTTCCCGAGGA

CGTTTCCCCGGGGCAGATCCTCCTTCTGGACGACGGCCGCATCCGGCTCAAGGTCCTGGAGGTCCGGAGC

CCGGAGATCCTCACGGAGGTGGAGGTGGGCGGGGTCCTCTCCAACAACAAGGGGATCAACATCCCTGGGG

CGGACCTCTCCATCCCCGCCCTCTCGGAGAAGGATATCCAGGACCTGGCCCTGGGGGCGGAGCTCGGGGT

GGACTGGGTGGCGGTCTCCTTCGTCCGCACGCGGGACGACCTCCTCCTCGCCCGGCACTACCTCTCCCGC

TACGGCTCCAAGGCCAGGCTCATGGCCAAGATCGAGAAGCCCTCGGCCGTGGCCCGGTTTGAGGAGATCC

TGGAGGAGGCGGACGGGATCATGGTGGCCCGGGGGGACCTCGGGGTGGAGATGCCCCTGGAGGAGGTGCC

CATCGTCCAGAAGCGCCTCATCCTCCGGTGCATCGCCGCCGGGAAGCCGGTGATCACCGCCACCCAGATG

CTGGAGTCCATGGTGCAGAACCCGAGCCCCACCCGGGCCGAGGCCTCGGACGTGGCCAACGCCATCTTTG

ACGGGACCGACGCGGTGATGCTCTCCGCGGAGACGGCGGCCGGGGCCTACCCGGTGGAGGCGGTGGCCAT

GATGGCGAGGATCGCCAAGGCGGTGGAGTCTTCCCCGGAGTTCTTGCAGAAGCTCAACGTCCTCCGCCCC

GCCCCCACCCCCACCACCCAGGACGCCATCGCCCAGGCGGCGGACGACGTGGTGGAGGCGGTGGGGGCCC

GGGCCATCGTGGTCTTCACGGCCACGGGCGGCTCGGCGCGGAGGATCGCCCGCACCCGGCCCCAGGTGCC

CATCCTGGCCCTCACCCCGAACCCCGAGGTGCGGAACCAGCTGGCCTTGGTCTGGGGGGTCTACCCCCAT

CTCGCCCCCGACCCCCAGGACACCGACGACATGGTCCGCATCGCCCTCAGGGAGGTGAAGGCCCTGGGGC

TCGCCCAGGTGGGGGACCGGGTGGTCATCGCCGCCGGGGTTCCCTTCGGGGTCCGGGGGACGACGAACCT

CATCCGCGTGGAGCGGGTGGGGTAa

**Designed Acetolactate synthase (BsAlsS-P)** *this study

**ATG**GGCAGCAGC**CATCATCATCATCATCAC**AGCAGCGGC**CTGGTGCCGCGCGGCAGCCAT**ATGCTTACCAAAGCAACCAAGGAGCAGAAGTCTTTGGTGAAGAACCGTGGAGCAGAACTTGTAGTTGACTGTCTTATCAATCAAGGCGTGACCCATGTCTTCGGGATCCCTGGTGCGAAGATCGACGCCGTTTTTGATGCTCTTCAAGACAAGGGACCAGAGATCATCGTGGCGCGCCACGAACAGAATGCGGCCTTCATGGCCCAAGCAGTCGGCCGTTTAACCGGCAAGCCTGGAGTCGTACTTGTAACGTCGGGACCTGGGGCTAGCAATTTAGCCACCGGCCTTCTGACCGCGAACACCGAGGGCGATCCGGTCGTGGCGTTGGCAGGGAATGTTCCTCGCGCAGATCGCCTTAAGCGCACGCACCAGTCCTTAGACAACGCCGCCCTTTTCCAGCCTATTACCAAGTACTCGGTCGAAGTTCAGGATCCTGACAATATCCCGGAAGCCGTAACGAACGCCTTCCGCATCGCGGAGGCAGGACAAGCCGGGGCGGCCTTTGTGTCCTTTCCCCAAGATGTTGTAAATGAAGTGACCAACACCAAAAATGTACGTCCTGTGGCGCCACCGAAGTTAGGTCCGGCTCCAGATGATGCCATCTCCGCTGCCATTGCAAAAATTCAAACGGCTAAGTTGCCGGTCGTTTTAGTAGGTATGAAGGGTGGCCGTCCAGAAGCCATTAAGGCCATTCGTAAGCTTTTGAAAAAAACACAATTACCCTTTGTGGAGACTTATCAGGCGGCTGGAACTTTGAGTCGTGACTTAGAGGATTTGTATTTTGGACGCATTGGCCTGTTCCGTAATCAGCCGGGTGATCTGCTTTTAGAACAGGCTGATGTAGTGTTGACGATCGGATATGATCCGATTGAGTATGATCCTAAATTTTGGAATATTAATGGAGACCGTACTATTATTCATTTAGACGAAATTCCTGCCGACATTGATCACTACTACCAACCAGACTTAGAATTAATTGGCGACATTCCATCAACGATCAACCATATTGCACACGACGCTGTGAAAGTCGAGTTTTCAGAGCGTGAACAAAAAATTCTGTCCGACCTGAAACAGTATATGCATGAACTGGAACAGGTACCTGCTGATTGGAAGAGCGACCGTGCACACCCCTTGGAAATTGTGAAAGAGTTACGCAACGCGGTGGATGATGACGTTACTGTTACATGTGACATCGGGTCGCACGCTATCTGGATGGCCCGCTACTTTCGCAGCTACGAGCCGCTGACTCTTATGATCTCTAATGGTATGCAAACCCTGGGTGTTGCTCTTCCATGGGCTATCGGCGCCTCTTTGGTAAAGCCGGGAGAGAAAGTTGTTTCGGTTTCGGGCGACGGGGGGTTCTTGTTCAGTGCTATGGAACTTGAGACTGCCGTCCGTTTGAAAGCGCCCATCGTGCATATCGTCTGGAATGATTCAACTTACGACATGGTAGCGTTCCAACAATTAAAAAAGTACAACCGTACCTCAGGGGTTGATTTCGGAAACATTGATATTGTCAAGTATGCGGAATCGTTTGGAGCAACGGGCTTACGTGTTGAGAGCCCCGACCAACTGGCGGACGTTTTGCGCCAGGGCATGAATGCTGAGGGTCCAGTCATTATCGACGTCCCCGTGGACTATTCTGATAATATCAACCTGGCAAGTGACAAGCTTCCTAAGGAGTTCGGTGAGCTGATGAAAACTAAGGCATTG**TAA**

**Ketol-acid reductoisomerase from *Geobacillus stearothermophilus* (GsIlvC)**

ATGGGCAGCAGCCATCATCATCATCATCACAGCAGCGGCCTGGTGCCGCGCGGCAGCCATATG

GCAAAAGTCTACTATAACGGGGATGCAAACGAACAATATTTGCAAGGGAAAACGGTCGCGATCATCG

GCTACGGCTCACAAGGCCACGCCCATGCACAAAACTTGCGCGACAGCGGCGTCCGCGTCATTGTCGGGTT

GCGCAAAGGGAAATCGTGGGAGCAGGCGGAACAGGACGGCTTTGAAGTATACTCGGTGCGCGAAGCGGCA

AAACAAGCGGATATTGTGATGGTGTTGCTGCCGGATGAGAAACAGCCGGCTGTCTACAAGGAAGAGATTG

AACCGGGGCTTGAACCGGGCAACGCGCTTGTGTTTGCGCACGGGTTTAACATTCATTTCAGCCAAATCGT

CCCGCCGGAGCATGTCGATGTCTTCTTGGTGGCGCCGAAAGGACCAGGCCATCTCGTGCGTCGCACATAT

GCGGAAGGAGCCGGGGTGCCGGCGCTCATCGCCGTTTATCAAGATGTAACCGGACACGCGAAAGAAACGG

CGCTCGCCTATGCGAAAGCGATTGGTGCTGCTCGAGCTGGGGTGTTGGAGACAACGTTCAAAGAAGAGAC

GGAAACCGACTTGTTCGGTGAACAAGCGGTGCTGTGCGGCGGGCTGACGGCGCTCATCAAGGCCGGGTTT

GAAACGCTCGTTGAAGCCGGGTATCAGCCGGAAGTCGCCTATTTCGAGTGTTTGCATGAAATGAAGCTCA

TCGTTGATCTTCTTTATGAAGGCGGCTTGTCGTGGATGCGCTACTCGATTTCCGATACGGCGCAATGGGG

CGACTTTATCACCGGTCCGCGCATCATTAACGACGCCGTGAAAGCGGAAATGAAAAAGGTGCTCGATGAC

ATCCAAACCGGCAAATTCGCGAAAAGCTGGATTTTGGAAAACCAAGCAAACCGTCCGGAGTTCAACGCCA

TCAACCGGCGCGAGAACGAGCATTTAATCGAAGTCGTCGGACGCGAACTGCGGAGCATGATGCCGTTTGT

GAAAGCAAAACAAAAAGAAGCGGTGGTGCCAGGTGCGAAACATTAA

**Dihydroxyacid dehydratase from *Streptococcus mutans* (SmIlvD)**

ATGGGCAGCAGCCATCATCATCATCATCACAGCAGCGGCCTGGTGCCGCGCGGCAGCCATATG

AAAGGAAGCGGTCAAATGACTGACAAAAAAACTCTTAAAGACTTAAGAAATCGTAGTTCTGTTTACG

ATTCAATGGTTAAATCACCTAATCGTGCTATGCTGCGTGCAACTGGTATGCAAGATGAAGACTTTGAAAA

ACCTATCGTCGGTGTCATTTCAACTTGGGCTGAAAACACACCTTGTAATATCCACTTACATGACTTTGGT

AAACTAGCCAAAGTCGGTGTTAAGGAAGCTGGTGCTTGGCCAGTTCAGTTCGGAACAATCACGGTTTCTG

ATGGAATCGCCATGGGAACCCAAGGAATGCGTTTCTCCTTGACATCTCGTGATATTATTGCAGATTCTAT

TGAAGCAGCCATGGGAGGTCATAATGCGGATGCTTTTGTAGCCATTGGCGGTTGTGATAAAAACATGCCC

GGTTCTGTTATCGCTATGGCTAACATGGATATCCCAGCCATTTTTGCTTACGGCGGAACAATTGCACCTG

GTAATTTAGACGGCAAAGATATCGATTTAGTCTCTGTCTTTGAAGGTGTCGGCCATTGGAACCACGGCGA

TATGACCAAAGAAGAAGTTAAAGCTTTGGAATGTAATGCTTGTCCCGGGCCTGGAGGCTGCGGTGGTATG

TATACTGCTAACACAATGGCGACAGCTATTGAAGTTTTGGGACTCAGCCTTCCGGGTTCATCTTCTCACC

CGGCTGAATCCGCAGAAAAGAAAGCAGATATTGAAGAAGCTGGTCGCGCTGTTGTCAAAATGCTCGAAAT

GGGCTTAAAACCTTCTGACATTTTAACGCGTGAAGCTTTTGAAGATGCTATTACTGTAACTATGGCTCTG

GGAGGTTCAACCAACTCAACCCTTCACCTCTTAGCTATTGCCCATGCTGCTAATGTGGAATTGACACTTG

ATGATTTCAATACTTTCCAAGAAAAAGTTCCTCATTTGGCTGATTTGAAACCTTCTGGTCAATATGTATT

CCAAGACCTTTACAAGGTCGGAGGGGTACCAGCAGTTATGAAATATCTCCTTAAAAATGGCTTCCTTCAT

GGTGACCGTATCACTTGTACTGGCAAAACAGTCGCTGAAAATTTGAAGGCTTTTGATGATTTAACACCTG

GTCAAAAGGTTATTATGCCGCTTGAAAATCCTAAACGTGAAGATGGTCCGCTCATTATTCTCCATGGTAA

CTTGGCTCCAGACGGTGCCGTTGCCAAAGTTTCTGGTGTAAAAGTGCGTCGTCATGTCGGTCCTGCTAAG

GTCTTTAATTCTGAAGAAGAAGCCATTGAAGCTGTCTTGAATGATGATATTGTTGATGGTGATGTTGTTG

TCGTACGTTTTGTAGGACCAAAGGGCGGTCCTGGTATGCCTGAAATGCTTTCCCTTTCATCAATGATTGT

TGGTAAAGGGCAAGGTGAAAAAGTTGCCCTTCTGACAGATGGCCGCTTCTCAGGTGGTACTTATGGTCTT

GTCGTTGGTCATATCGCTCCTGAAGCACAAGATGGCGGTCCAATCGCCTACCTGCAAACAGGAGACATAG

TCACTATTGACCAAGACACTAAGGAATTACACTTTGATATCTCCGATGAAGAGTTAAAACATCGTCAAGA

GACCATTGAATTGCCACCGCTCTATTCACGCGGTGTCCTTGGTAAATATGCTCACATCGTTTCGTCTGCT

TCTAGGGGAGCCGTAACAGACTTTTGGAAGCCTGAAGAAACTGGCAAAAAATaA

**KivD-S (**This study)

ATGGGCAGCAGCCATCATCATCATCATCACAGCAGCGGCCTGGTGCCGCGCGGCAGCCATATGTATACTGTTGGGGACTACTTGTTGGACCGCTTACATGAGTTAGGAATCGAACATATCTTTGGCGTTCCCGGCGACTACAACCTTCAATTTTTAGACCACATCATCAGTCGTAAAGATATGAAATGGGTCGGGAACGCGAATGAGTTGAATGCGAGTTACATGGCTGATGGGTATGCCCGCACGAAGAAAGCGGCTGCTTTCCTGACGACATTCGGCGTCGGTGAGCTTTCTGCGGTGAATGGGCTGGCGGGGAGCTACGCGGAGAATCTTCCCGTTGTGGAGATTGTTGGCTCACCCACCTCGAAGGTTCAAAATGAAGGCAAATTCGTCCATCACACCTTGGCGGATGGAGATTTTAAACACTTTATGAAGATGCACGAGCCGGTGACGGCAGCGCGCACATTATTAACAGCAGAGAATGCAACAGTAGAGATTGATCGCGTTTTATCCGCTCTGTTAAAGGAGCGCAAGCCCGTCTATATTAATCTGCCTGTTGATGTCGCGGCCGCTAAAGCGGAAAAACCTTCCCTGCCGCTGAAAAAAGAAAATAGCACGAGTAATACGTCAGATCAGGAGATCTTAAATAAGATCCAAGAATCGTTAAAAAACGCCAAAAAGCCAATCGTTATCACCGGGCACGAGATTATCAGCTTTGGGTTGGAAAAAACTGTGAGTCAATTTATCTCTAAGACCAAACTTCCCATCACCACGTTAAATTTCGGGAAGTCCTCTGTGGATGAATCACTGCCCAGTTTCTTGGGAATTTATAATGGCAAGCTTTCTGAGCCTAATTTGAAGGAATTCGTAGAATCAGCAGATTTTATCCTGATGCTTGGGGTGAAGCTTACAGACTCCAGTACAGGCGTATTTACGCATCACCTTGACGAAAATAAGATGATCTCGCTGAACATCGATGAGGCGAAAATCTTCGGGGAGTCGATCCAGAACTTTGATTTCGAGTCTCTTATTAAATCTCTGCTGGACTTGAGTGAGATTGAGTATAAGGGAAAATACATCGATAAAAAGCAGGAGGACTTCGTGCCCAGTAATGCGTTACTGTCGCAAGATCGTCTGTGGCAGGCTGTCGAGCATTTAACGCAATCCAATGAGGTCATTGTCGCTGAGCAGGGAACCTCTTTCTTCGGCGTGTCCCCTATTCGCTTGAAACCAGGATCGACGTTTATTGGACAACCCTTGTGGGGCTCGATTGGATATACATTCCCGGCCGCTTTGGGTTCGCAAATTGCAGACCCCGAGCGCCGCCATATCCTGTTTATTGGGGATGGAAGCCTGCAAATGTCTGTCCAAGAGTTAGGGTTGGCCATTCGCGAAGGGATTAATCCCATTATCTTCATTATCAATAACGATGGATATACTGTCGAACGCGAGATTCACGGGCCGAATCAAAGTTACAACGACATTCCAATGTGGAACTATAGCAAGCTTCCCGAGAGCTTCGGGGCGACTGAAGAACGTGTCGTTTCAAAAATTGTAACAACCGAGAACGAGTTCGTAAGTGTGATGAAGGAAGCCCAGGCTGACCCGAACCGCATGTATTGGATCGAACTTATCTTAGCTAAGGAGGACGCACCAAAAGTTCTGAAAAAAATGGGTAAGTTGCTGGCCGAACAAAATAAGTAA

**Aldehyde reductase from *Escherichia coli* (EcYahK)**

ATGGGCAGCAGCCATCATCATCATCATCACAGCAGCGGCCTGGTGCCGCGCGGCAGCCATATG

AAGATCAAAGCTGTTGGTGCATATTCCGCTAAACAACCACTTGAACCGATGGATATCACCCGGCGTGAACCGGGACCGAATGATGTCAAAATCGAAATCGCTTACTGTGGCGTTTGCCATTCCGATCTCCACCAGGTCCGTTCCGAGTGGGCGGGGACGGTTTACCCCTGCGTGCCGGGTCATGAAATTGTGGGGCGTGTGGTAGCCGTTGGTGATCAGGTAGAAAAATATGCGCCGGGCGATCTGGTCGGTGTCGGCTGCATTGTCGACAGTTGTAAACATTGCGAAGAGTGTGAAGACGGGTTGGAAAACTACTGTGATCACATGACCGGCACCTATAACTCGCCGACGCCGGACGAACCGGGCCATACTCTGGGCGGCTACTCACAACAGATCGTCGTTCATGAGCGATATGTTCTGCGTATTCGTCACCCGCAAGAGCAGCTGGCGGCGGTGGCTCCTTTGTTGTGTGCAGGGATCACCACGTATTCGCCGCTACGTCACTGGCAGGCCGGGCCGGGTAAAAAAGTGGGCGTGGTCGGCATCGGCGGTCTGGGACATATGGGGATTAAGCTGGCCCACGCGATGGGGGCACATGTGGTGGCATTTACCACTTCTGAGGCAAAACGCGAAGCGGCAAAAGCCCTGGGGGCCGATGAAGTTGTTAACTCACGCAATGCCGATGAGATGGCGGCTCATCTGAAGAGTTTCGATTTCATTTTGAATACAGTAGCTGCGCCACATAATCTCGACGATTTTACCACCTTGCTGAAGCGTGATGGCACCATGACGCTGGTTGGTGCGCCTGCGACACCGCATAAATCGCCGGAAGTTTTCAACCTGATCATGAAACGCCGTGCGATAGCCGGTTCTATGATTGGCGGCATTCCAGAAACTCAGGAGATGCTCGATTTTTGCGCCGAACATGGCATCGTGGCTGATATAGAGATGATTCGGGCCGATCAAATTAATGAAGCCTATGAGCGAATGCTGCGCGGTGATGTGAAATATCGTTTTGTTATCGATAATCGCACACTAACAGACTaA
